# A Case of Hepatosplenic Gamma Delta T Cell Lymphoma With Concomitant Bone Marrow Aplasia Following Azathioprine Therapy, Treatment Course and Review of the Literature

**DOI:** 10.1002/cnr2.70334

**Published:** 2025-09-04

**Authors:** Rania Younis, Holger Hauspurg, Andreas Voss

**Affiliations:** ^1^ Department of Oncology and Haematology Klinikum Oldenburg and Faculty of Medicine, University of Oldenburg Oldenburg Germany; ^2^ Institute for Hematopathology Hamburg Hamburg Germany

**Keywords:** allogeneic stem cell transplantation, aplastic anemia, azathioprine, bone marrow failure, gamma‐delta T cells, hepatosplenic T‐cell lymphoma, immunosuppressive therapy

## Abstract

**Background:**

Hepatosplenic T‐cell lymphoma (HSTCL) is a rare and aggressive subtype of peripheral T‐cell lymphoma with a poor prognosis, primarily affecting young adult males, many with a background of immunosuppression or autoimmune disease.

**Case:**

We present the case of a 27‐year‐old male previously treated with azathioprine who developed pancytopenia. Bone marrow biopsy revealed severe aplasia with partial infiltration by gamma‐delta T‐lymphocytes (Tγδ). A definitive diagnosis of HSTCL was established through liver biopsy. The patient received induction chemotherapy with the Ifosfamide, Carboplatin, Etoposide (ICE regimen) but remained pancytopenic, prompting allogeneic stem cell transplantation. Sixty months post‐treatment, he remains in complete remission.

**Conclusion:**

To our knowledge, this is the first reported case of HSTCL presenting concurrently with aplastic anemia in the context of prior azathioprine exposure. The underlying pathophysiology is likely multifactorial, involving both immune‐mediated suppression and drug‐induced toxicity. Clinicians should maintain a high index of suspicion for lymphoma in patients developing unexplained pancytopenia while on azathioprine therapy.

## 1 Introduction

Gamma‐delta T cells (Tγδ) are a small but functionally significant subset of T lymphocytes derived from double‐negative (CD4^−^CD8^−^) thymocytes in the bone marrow [1]. Unlike the predominant αβ T‐cell population, Tγδ cells express a unique T‐cell receptor composed of gamma and delta chains, enabling them to recognize a wide range of antigens independently of major histocompatibility complex molecules [2]. Two major subsets are defined by their delta chain usage: Vδ1 T cells, found primarily in the thymus and peripheral tissues, and Vδ2 T cells, which dominate in the blood [3]. Both subsets exhibit cytotoxic potential mediated by γδ TCR and natural killer (NK) group 2D receptors through the release of soluble mediators [4].

Tγδ cells play an essential role in immune responses due to their cytotoxicity and pro‐inflammatory profile [2]. Their involvement has been documented in autoimmune diseases, chronic inflammatory conditions [3], and various malignancies, where they may exhibit both tumor‐promoting and tumor‐suppressing functions [4, 5, 6]. Hepatosplenic T‐cell lymphoma (HSTCL) aggressive malignancy arising from this cell type; it is a rare neoplasm accounting for less than 1% of all lymphoid tumors [7]. HSTCL typically affects young adults, with a male predominance [8]. Its pathogenesis is believed to involve chronic antigenic stimulation and immune dysregulation [9], although most reported cases occur in immunocompetent individuals [8, 10].

Approximately 18% of HSTCL cases occur in immunosuppressed patients, often with underlying autoimmune diseases, inflammatory bowel disease (IBD), or prior organ transplantation [8, 11]. Exposure to immunosuppressive agents—particularly azathioprine (AZA) and infliximab—has been frequently reported in this context. Although viral associations, such as Epstein–Barr virus and hepatitis B or C, have been noted in isolated cases, the relationship remains unclear [12].

Histologically, HSTCL is characterized by a diffuse sinusoidal infiltration of medium‐sized lymphocytes in the spleen, liver, and bone marrow, while lymph node involvement is rare [13, 14]. The neoplastic cells are typically of γδ TCR origin, often from the Vδ1 subset, though 5%–20% of cases express an αβ TCR phenotype [1, 15]. Immunophenotypically, the cells are double‐negative for CD4 and CD8, and positive for CD2, CD3, CD7, and TCRγδ, with occasional expression of NK‐associated markers such as CD16, CD56, and CD57 [16, 17]. The most consistent cytogenetic finding is isochromosome 7q [i(7q)] [18], with other aberrations such as trisomy 8 and Y chromosome loss reported [19].

Due to its rarity, standardized treatment guidelines for HSTCL are lacking. CHOP‐based regimens remain commonly used but yield poor response rates [11]. More intensive regimens like Ifosfamide, Carboplatin, Etoposide (ICE) or Ifosfamide, Etoposide, high‐dose Cytarabine (IVAC) have shown improved outcomes in small cohorts [20, 21, 22]. Both autologous and allogeneic stem cell transplantations (SCTs) have been used as a modality of treatment with a more favorable outcome. Allogeneic SCT has demonstrated the most promising results, with some patients achieving long‐term survival and a reported median overall survival of 59 months post‐transplant [21]. Treatment of relapsed disease remains particularly challenging, with high treatment‐related morbidity and limited success.

AZA, a 6‐mercaptopurine derivative, is widely used in managing autoimmune diseases and preventing organ transplant rejection [23, 24]. While reversible bone marrow suppression is a known side effect [25], rare but serious complications, including aplastic anemia and persistent marrow failure, have been documented [26, 27, 28]. An increased incidence of lymphomas, including HSTCL, has also been associated with long‐term AZA use, particularly in patients with IBD [24, 29, 30]. A UK population‐based study, for example, found that patients with IBD treated with AZA had a significantly higher risk of developing lymphoma, with an odds ratio (OR) of 3.22 compared to controls [29].

In this report, we describe a rare case of hepatosplenic T‐cell lymphoma presenting concurrently with severe aplastic anemia in a young adult previously treated with AZA. Although both bone marrow suppression and lymphoma are known risks of AZA therapy, their co‐occurrence is exceedingly rare and diagnostically challenging. To our knowledge, this is the first reported case of HSTCL manifesting with persistent bone marrow aplasia in the context of prior AZA exposure. This case underscores the importance of considering both lymphoproliferative disorders and acquired aplastic anemia in patients with persistent cytopenia while on immunosuppressive therapy, and highlights the need for timely diagnostic evaluation and aggressive treatment in such scenarios.

## 2 Case Presentation

A 27‐year‐old male with a significant past medical history of leukocytoclastic vasculitis (previously treated with AZA), *Clostridium difficile* colitis with small bowel obstruction, and chronic tonsillitis presented to the emergency department of our hospital (Klinikum Oldenburg, Germany) in February 2020 with general malaise, a 1‐week history of fever, approximately 4 kg of weight loss over 2 weeks, and petechiae on the lower extremities. Physical examination was unremarkable except for the presence of petechiae.

### 2.1 Investigations

Initial laboratory tests revealed pancytopenia: hemoglobin 9.3 g/dL (14.0–18.0), white blood cell count 1.5 × 10^9^/L (4.0–10.0), and platelet count 3 × 10^9^/L (150–450). C‐reactive protein was markedly elevated at 15.2 mg/dL (< 0.5), and lactate dehydrogenase was mildly elevated at 280 U/L. Renal and liver function tests were within normal limits. Serological testing was negative for hepatitis A, B, C, HIV, and CMV. EBV‐EBNA‐IgG and EBV‐VCA‐IgG were positive, while EBV‐IgM was negative, indicating past exposure. Screening for autoimmune and vasculitic disorders was unremarkable. Laboratory testing also revealed deficiencies in vitamin B12 (112 ng/L [230–970]) and folic acid (2.5 μg/L [4.6–27.]).

Abdominal ultrasound and cross‐sectional imaging (CT and MRI) demonstrated hepatosplenomegaly and multiple mildly enlarged lymph nodes in the submandibular, cervical, and para‐aortic regions, with the largest measuring 1.2 × 0.9 cm. A suspicious lesion was also noted in the left upper lobe of the lung.

**FIGURE 1:**
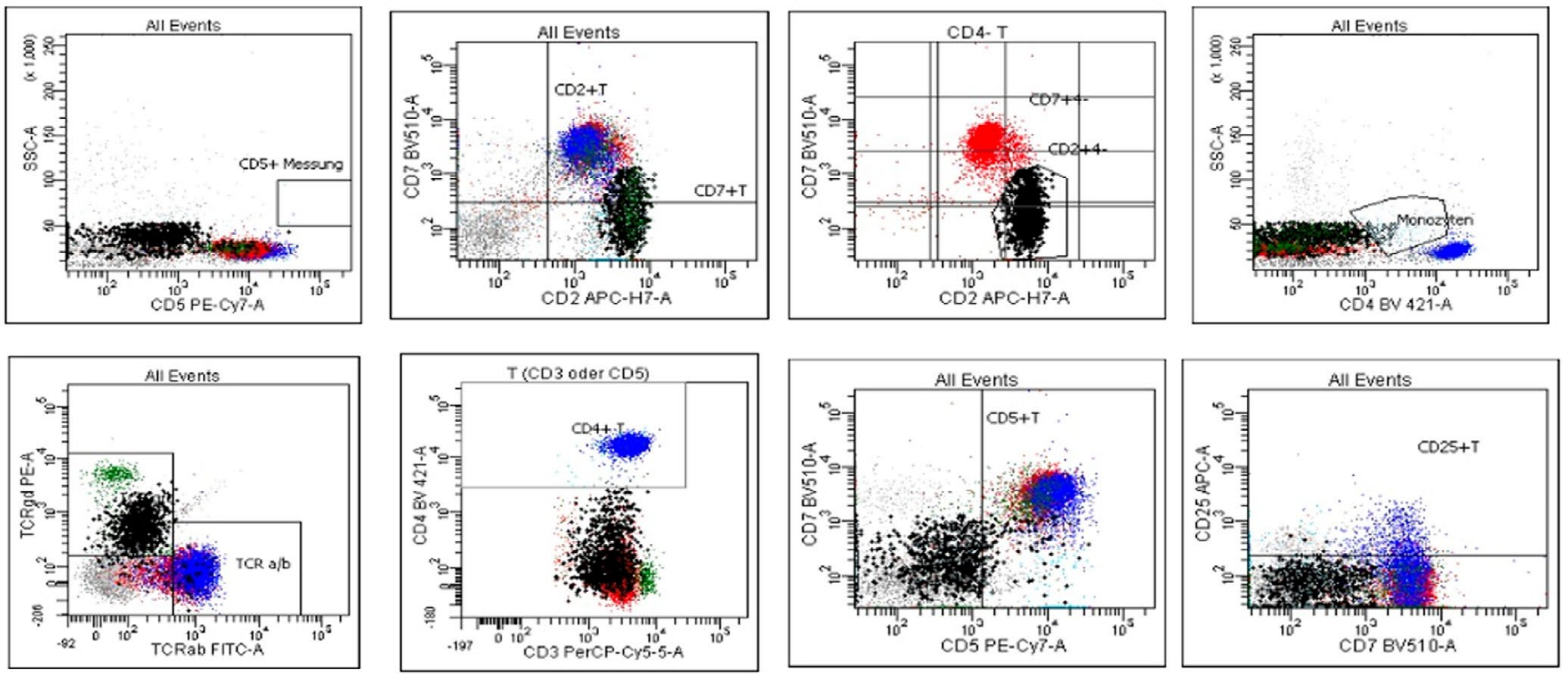
Flow cytometry analysis of bone marrow aspirate. Multicolor flow cytometry of the bone marrow from a 27‐year‐old patient with hepatosplenic T‐cell lymphoma (HSTCL). The aberrant cell population is highlighted in black across the dot plots. Analysis shows: A CD2^+^CD3^+^CD4^−^CD5^−^CD7^+^ T‐cell phenotype with negative CD5 expression. Expression of T‐cell receptor γ/δ (TCRγδ) and absence of TCRα/β, confirming γδ T‐cell lineage.

Bone marrow aspiration and biopsy revealed a hypocellular, aplastic marrow across all hematopoietic lineages. This was initially attributed to prior AZA exposure. Flow cytometry identified a population of T cells expressing γδ T‐cell receptors. Immunohistochemistry revealed cells positive for CD2 and NK markers CD16 and CD56, but negative for CD4, CD5, CD7, CD8, and CD57—consistent with bone marrow infiltration by HSTCL as shown in Figure 1.

**FIGURE 2:**
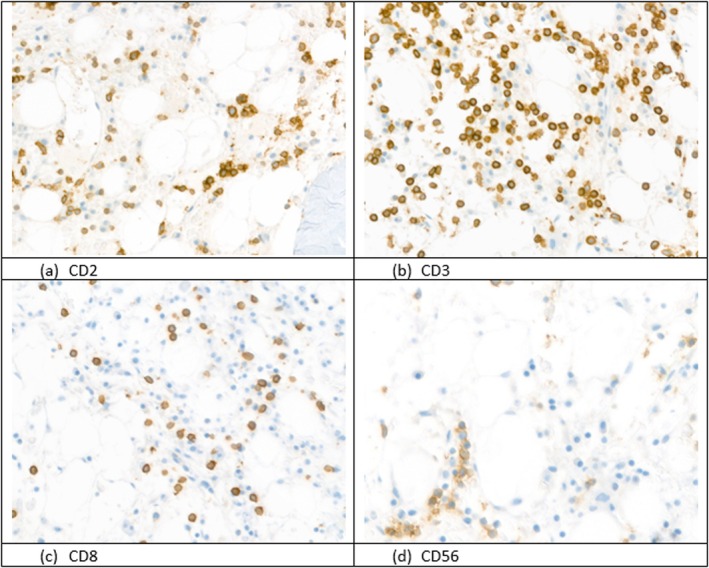
Immunohistochemical staining of bone marrow biopsy (original magnification ×400). Bone marrow trephine biopsy from a 27‐year‐old patient with hepatosplenic T‐cell lymphoma (HSTCL), demonstrating: (a) CD2: Strong membranous and cytoplasmic positivity in infiltrating lymphoid cells, confirming pan‐T‐cell lineage. (b) CD3: Diffuse expression highlighting the T‐cell origin of the neoplastic infiltrate. (c) CD8: Negative staining in lymphoid cells. (d) CD56: Patchy membranous staining, supporting γδ T‐cell lineage, as commonly observed in HSTCL. All stains reveal interstitial and sinusoidal infiltration of the bone marrow. Blue nuclear counterstain (hematoxylin).

These γδ cells comprised approximately 11% of the total cell population and exhibited a sinusoidal distribution on bone marrow histology (Figure 2). Cytogenetic analysis demonstrated isochromosome 7q and trisomy 8.

**FIGURE 3:**
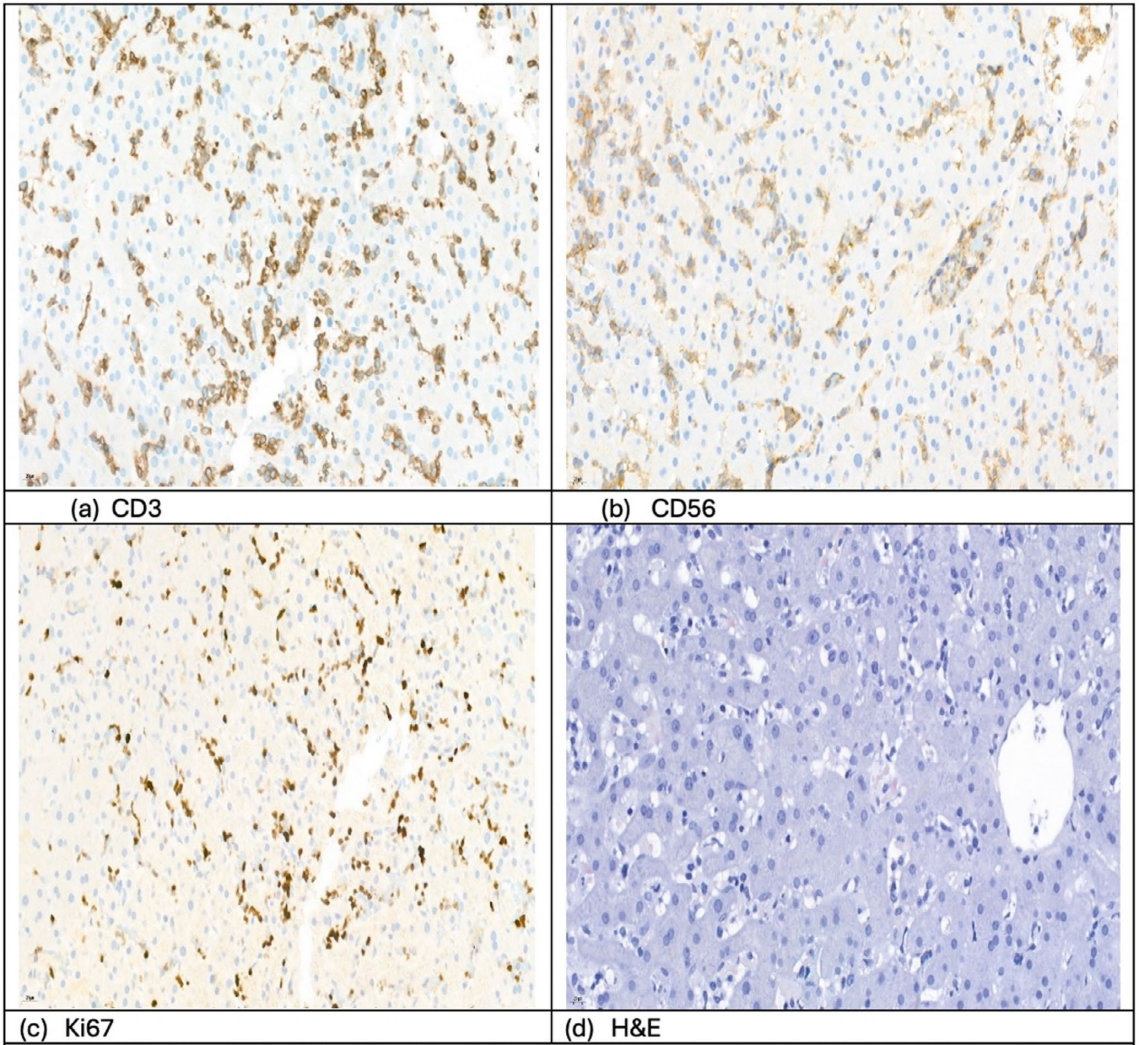
Histopathologic and immunohistochemical analysis of liver biopsy (original magnification ×400). Liver biopsy from a 27‐year‐old patient with hepatosplenic T‐cell lymphoma (HSTCL) showing: (a) CD3: Positive staining of sinusoidally distributed T lymphocytes. (b) CD56: Strong sinusoidal expression, consistent with γδ T‐cell lineage. (c) Ki‐67: High proliferative index in infiltrating lymphocytes, reflecting aggressive tumor biology. (d) Hematoxylin and eosin (H&E): Dense sinusoidal infiltration by atypical lymphoid cells with preserved hepatic architecture. These findings support the diagnosis of HSTCL with predominant sinusoidal involvement by cytotoxic T cells.

To confirm the diagnosis, an ultrasound‐guided liver biopsy was performed. This procedure was complicated by a subcapsular hematoma due to thrombocytopenia, despite prophylactic transfusions. The complication was managed conservatively with repeated transfusions of platelets, fresh frozen plasma, and clotting factors. Histological analysis of the liver biopsy confirmed infiltration by γδ T‐cells (Figure 3).

### 2.2 Treatment and Complications

**FIGURE 4:**
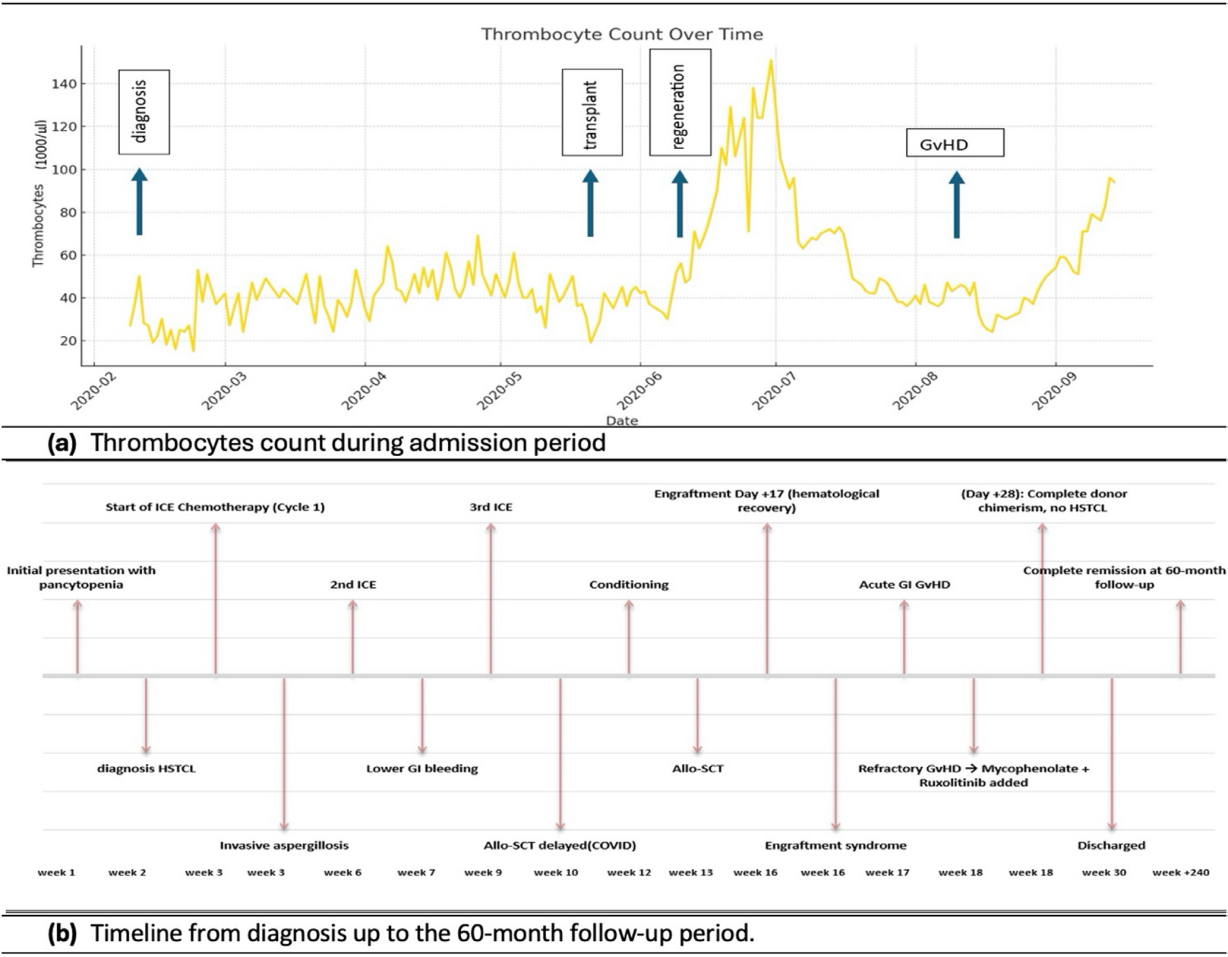
Thrombocyte count trend during the admission period and clinical timeline from initial diagnosis to report completion. (a) Thrombocyte count during admission period. (b) Timeline from diagnosis up to the 60‐month follow‐up period. (c) Line graph showing daily thrombocyte (platelet) counts (1000/μL) in the patient throughout the admission period. Despite radiologic and clinical partial response to ICE chemotherapy, the patient remained persistently thrombocytopenic, with platelet levels consistently below normal. No recovery of thrombocyte counts was observed prior to allogeneic stem cell transplantation. A total of 74 platelet bags have been transfused throughout the admission period. (d) Chronological overview of the patient's clinical course, beginning at the time of diagnosis (02/2020) and extending through treatment phases up to the preparation of this report (02/2025). The timeline illustrates key events including diagnosis, induction chemotherapy (ICE), allogeneic stem cell transplantation (allo‐SCT), and post‐transplant follow‐up milestones.

Due to persistent bone marrow suppression, the patient was started on granulocyte colony‐stimulating factor. Following confirmation of the diagnosis, he received pre‐phase therapy with prednisolone and vincristine, followed by induction chemotherapy with the ICE regimen. He completed three cycles at three‐week intervals but failed to achieve hematologic recovery. Figure 4a illustrates the persistent thrombocytopenia during the therapy.

**FIGURE 5:**
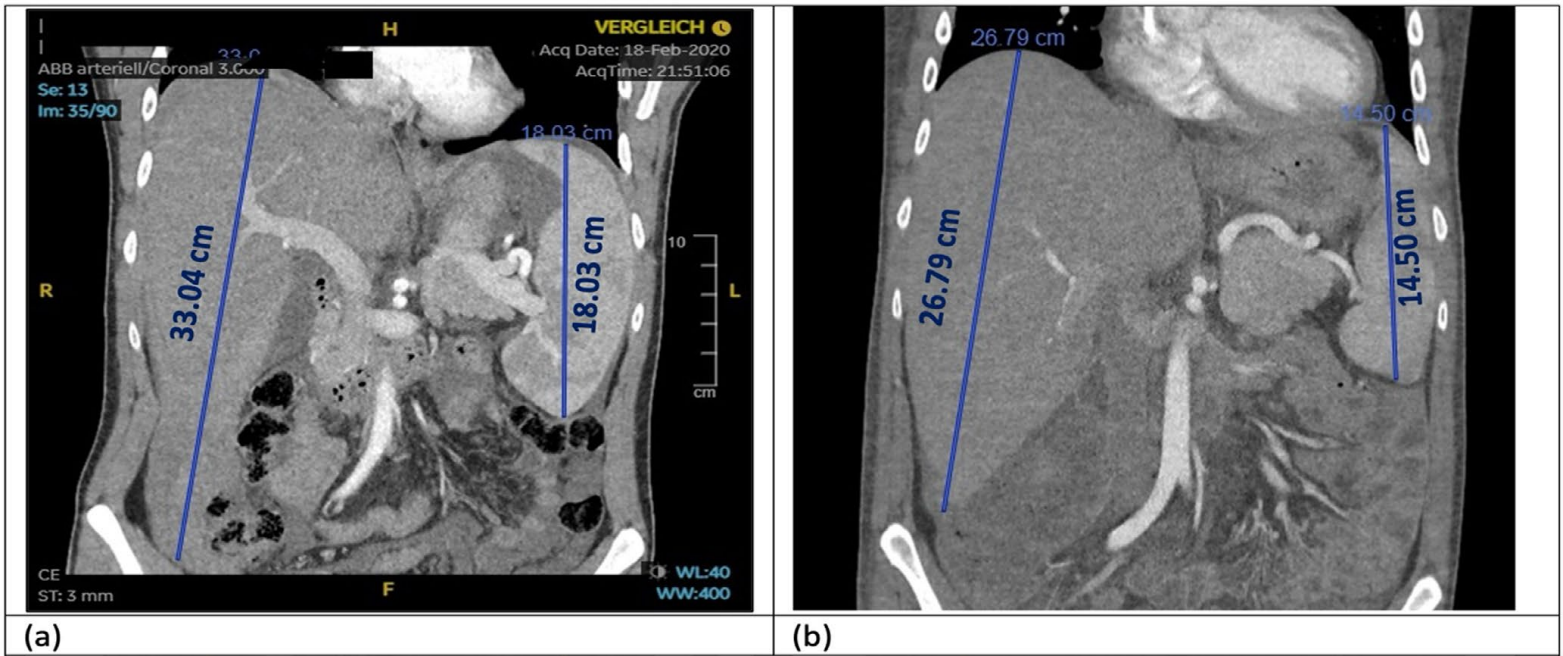
Coronal abdominal CT images of a 27‐year‐old patient with hepatosplenic T‐cell lymphoma (HSTCL). (a) CT at time of diagnosis (February 2020) showing significant hepatosplenomegaly with the liver measuring 33.04 cm and the spleen 18.03 cm in craniocaudal extension. (b) Follow‐up CT after 3 cycles of ICE chemotherapy (May 2020, prior to allogeneic stem cell transplantation), demonstrating a notable reduction in organ size: the liver measures 26.79 cm and the spleen measures 14.5 cm, indicating a partial response to induction therapy.

During induction, he developed neutropenic fever with sepsis. Thoracic CT imaging revealed signs of invasive pulmonary aspergillosis, for which antifungal therapy with voriconazole was initiated. Restaging after two ICE cycles showed partial remission (Figure 5) with reduction in hepatosplenomegaly and marrow infiltration. However, the patient remained profoundly cytopenic.

He also developed lower gastrointestinal bleeding, with endoscopic findings of an ulcer at the ileocecal junction, suspected to be due to lymphoma infiltration. Biopsy was not feasible due to thrombocytopenia. A repeat bone marrow biopsy showed persistent aplasia.

Given the poor prognosis and refractory pancytopenia, allogeneic SCT was planned. However, its initiation was delayed by COVID‐19‐related disruptions. Three months after diagnosis, following clinical stabilization, conditioning with Thiotepa, Fludarabine, and Treosulfan was commenced, followed by allogeneic SCT from a matched unrelated donor.

The patient achieved hematologic engraftment by day +17. Shortly after, he developed fever, weight gain, and respiratory insufficiency—symptoms interpreted as engraftment syndrome—which resolved with high‐dose prednisolone. Subsequently, he developed profuse watery diarrhea (> 1500 mL/day) and was diagnosed with clinical stage 4 acute gastrointestinal graft‐versus‐host disease (aGvHD, Harris classification), which was confirmed histologically (Lerner grade 2). Initial therapy included prednisolone, cyclosporine, and oral budesonide. Due to refractoriness, treatment was escalated with mycophenolate mofetil and later ruxolitinib, following tapering of prednisolone.

During this period, the patient also developed an iatrogenic pseudoaneurysm of the left carotid artery after a central line insertion attempt, requiring surgical management. Gradually, symptoms improved, and he was discharged on parenteral nutrition and outpatient follow‐up.

### 2.3 Outcome and Follow‐Up

Bone marrow analysis on day +28 demonstrated complete donor chimerism with no residual HSTCL infiltration. Follow‐up CT and MRI scans confirmed complete remission with normalization of liver and spleen size. Immunosuppressive therapy was gradually tapered, and parenteral nutrition was discontinued after GvHD improvement.

At the time of writing—60 months post‐diagnosis—the patient remains in complete remission with sustained full donor chimerism. He has resumed full‐time employment, gotten married, and is planning to build a home. Figure 4b summarizes the clinical course from diagnosis to the preparation of this report, 60 months later.

## 3 Discussion

To our knowledge, this is the first reported case of HSTCL presenting concurrently with aplastic anemia at the time of diagnosis. Aplastic anemia is a form of bone marrow failure marked by hypocellularity and impaired hematopoiesis, caused by intrinsic stem cell defects or dysfunctional marrow microenvironment [31]. While congenital causes such as Fanconi anemia are well‐characterized [31, 32, 33], acquired aplastic anemia may be triggered by environmental toxins, infections, medications, or autoimmune conditions [31, 34, 35].

Pancytopenia is a known but nonspecific feature of HSTCL and is often attributed to marrow infiltration, immune suppression, or hypersplenism [1, 11, 17, 36]. In our case, the bone marrow was severely hypocellular, with only a limited population (~11%) of γδ T cells. This modest infiltration suggests that marrow failure has been driven more by immune‐mediated mechanisms than by direct tumor burden.

Immune‐mediated cytopenia has been previously reported in T‐cell lymphomas. Burg et al. [37] described a case of subcutaneous γδ T‐cell lymphoma associated with leukopenia and elevated interferon‐γ levels, despite no bone marrow infiltration. Similarly, Pizzi et al. [38] reported cases of HSTCL complicated by hemophagocytic lymphohistiocytosis, likely driven by proinflammatory cytokines produced by malignant T cells.

In our patient, long‐term AZA exposure may have further contributed to marrow failure. Several cases have linked AZA to severe, and in some instances fatal, aplastic anemia [27, 28]. Sherwood et al. [27] reported a case of fatal aplastic anemia in a young man with autoimmune hepatitis. Unlike previously documented HSTCL cases with hypocellularity that resolved after lymphoma treatment [20], our patient showed no hematologic recovery post‐ICE chemotherapy, indicating an overlapping mechanism of AZA‐induced marrow toxicity and HSTCL‐related suppression.

This supports a dual pathogenesis in our patient involving both underlying drug‐induced aplastic anemia and bone marrow suppression related to HSTCL. Clinicians should remain vigilant for underlying malignancies in patients with persistent pancytopenia on immunosuppressants like AZA. Additionally, a better understanding of HSTCL immunopathology is needed. Profiling cytokines and growth factors secreted by γδ T cells may elucidate mechanisms of marrow suppression and inform targeted interventions.

Table 1 summarizes key literature on the association between thiopurine exposure, aplastic anemia, and HSTCL. For improved clarity, the table has been revised to focus on this specific clinical intersection.

**TABLE 1:** Azathioprine/6‐MP in HSTCL, aplastic anemia, and bone marrow involvement.

Ref	Author/year	Agent	Condition	Findings	Bone marrow	Outcome
[13]	Vega et al., 2001	6‐MP	HSTCL (IBD)	HSTCL with sinusoidal marrow infiltration; 1 of 8 patients had 6‐MP exposure (IBD); others without immunosuppressive history	Sinusoidal γδ T‐cell infiltration, hypercellular	Died of aggressive disease
[20]	Krishnan et al., 2019	—	HSTCL with cytopenia	Cytopenia improved post‐ICE	Initially hypocellular, recovered	CR after ICE
[26]	Montgomery et al., 2022	Azathioprine	Aplastic anemia	Persistent marrow failure after AZA	Aplastic	Refractory AA
[27]	Sherwood & Jafri, 2020	Azathioprine	Aplastic anemia	Fatal AA in autoimmune hepatitis	Hypocellular	Fatal despite withdrawal
[28]	Yeter et al., 2013	Azathioprine	AA (SLE)	Severe AA in lupus patient	Aplastic	Supportive care required
[29]	Armstrong et al., 2010	Azathioprine	Lymphoma (IBD)	Increased lymphoma risk with AZA	Not specified	Elevated risk
[30]	Kandiel et al., 2005	AZA/6‐MP	HSTCL in IBD	Meta‐analysis: HSTCL risk ↑ in immunosuppressed	Not specified	Often fatal
[36]	Ochenrider et al., 2010	6‐MP	HSTCL (IBD)	HSTCL after 6‐MP monotherapy	30%–40% γδ T‐cell infiltration	Death 7 months post‐diagnosis
—	Younis et al., 2024 (this case)	Azathioprine	HSTCL + aplastic anemia	Concurrent HSTCL and AA after AZA	Hypocellular + partial γδ infiltration	CR after allo‐SCT, alive at FU 60 months

In this case allogeneic SCT was ultimately required to address both conditions, leading to full hematologic and clinical remission. HSTCL management remains challenging due to its rarity and aggressive nature. CHOP‐based regimens are generally inadequate, with response rates of only 30%–45% [11]. More intensive therapies such as ICE or IVAC have demonstrated improved remission rates in small series [20, 21, 22, 39, 40]. Voss et al. [21] observed better outcomes in patients treated with multi‐agent regimens, potentially due to greater lymphocyte depletion [41, 42].

Despite temporary responses to chemotherapy, durable remission typically requires allogeneic SCT. Retrospective data from the North American Peripheral T‐Cell Lymphoma Consortium reported that 63% of HSTCL patients responded to induction therapy, but sustained remission occurred predominantly in those who underwent allogeneic SCT, with a median overall survival of just 15.8 months [10].

Emerging treatments such as immunotherapies targeting γδ TCRs and allogeneic CAR‐T cells offer potential but remain experimental [43, 44]. Given the rarity and poor prognosis of HSTCL, early diagnosis and prompt referral for transplantation are crucial to improving patient outcomes.

## 4 Conclusion

HSTCL is a rare and aggressive lymphoma predominantly affecting young adult males and is associated with poor outcomes. This case highlights a unique and clinically significant presentation of HSTCL with concurrent aplastic anemia, likely exacerbated by prior azathioprine therapy. Our experience reinforces the importance of early diagnosis and aggressive treatment using intensive chemotherapy and allogeneic SCT.

Given the rarity of HSTCL and the absence of standardized guidelines, international collaboration and further research are essential to improve understanding of its pathophysiology and to establish evidence‐based treatment strategies.

## Author Contributions

**Rania Younis:** writing – original draft, writing – review and editing, supervision, validation. **Holger Hauspurg:** methodology, validation, visualization, supervision. **Andreas Voss:** conceptualization, investigation, writing – original draft, methodology, validation, visualization, writing – review and editing, project administration, supervision.

## Ethics Statement

Written informed consent was obtained from the patient for publication of this case and accompanying images. The case was managed according to institutional ethical standards and the Declaration of Helsinki.

## Conflicts of Interest

R.Y. declares no conflicts of interest. H.H. received travel support from Roche and Astra Zeneca; honoraria from Abbvie, Novartis, and MSD; a research grant from Novartis; and consultancy fees from hema.to and Scopio Labs. A.V. received travel support from Amgen; honoraria from JAZZ, BMS/Celgene, Amgen, Sobi, Servier, Novartis, Delbert, and Pfizer.

## Data Availability

Data sharing not applicable to this article as no datasets were generated or analysed during the current study.
